# Phenotypic and Physiological Changes Associated with Senescence in Stay-Green *Elymus sibiricus* and Germplasm Screening

**DOI:** 10.3390/plants15132047

**Published:** 2026-07-01

**Authors:** Wenhu Wang, Wenhui Liu, Kaiqiang Liu, Wen Li, Rui Wu, Xin Chen, Wei Hu, Huimin Duan, Guoling Liang

**Affiliations:** 1 Academy of Animal Science and Veterinary, Qinghai University, Xining 810016, China; wwh01112021@163.com (W.W.); 2002990027@qhu.edu.cn (W.L.); 2023990047@qhu.edu.cn (K.L.); lw@qhu.edu.cn (W.L.); wuruiqh@163.com (R.W.); chenxinbaafs@163.com (X.C.); scihw123@163.com (W.H.); dhm_80@163.com (H.D.); 2 Key Laboratory of Qinghai Province Superior Forage Germplasm in the Qinghai–Tibet Plateau, Xining 810016, China

**Keywords:** senescence, *Elymus sibiricus*, stay-green, phenotypic characteristics, physiological properties, germplasm resources

## Abstract

Early senescence of alpine pasture grass, manifested as rapid yellowing at the onset of autumn on the Qinghai–Tibet Plateau, constrains the sustainable development of grassland animal husbandry. Breeding stay-green forage germplasm is key to mitigating this problem. To identify superior stay-green germplasm and preliminarily elucidate the main drivers of senescence, we evaluated six stay-green lines of *Elymus sibiricus* with non-stay-green materials as controls. Fixed-site field observations were conducted for three consecutive years in Haiyan County, Qinghai Province. We quantified dynamic changes in phenotypic, photosynthetic, and physiological traits during senescence, applied mixed-effects models to identify factors associated with stay-green, and used the TOPSIS model for comprehensive evaluation. The results showed that plant height, green leaf area, chlorophyll content, net photosynthetic rate, and root activity of stay-green *E. sibiricus* were significantly higher than those of non-stay-green materials at all planting years, and the senescence rate was significantly slower. All traits performed optimally at the third year. Relative to HB-2, HB-4, HB-8, HB-10, HB-11, and HB-15 (stay-green *E. sibiricus*), plant height, green leaf area, chlorophyll content, net photosynthetic rate, and root activity of CK (non-stay-green *E. sibiricus*) were 0.83, 0.95, 0.79, 0.82, 0.80, and 0.78; 0.37, 0.37, 0.34, 0.35, 0.31, and 0.35; 0.82, 0.84, 0.80, 0.86, 0.82, and 0.75; 0.86, 0.86, 0.74, 0.89, 0.77, and 0.70; and 0.72, 0.74, 0.66, 0.78, 0.70, and 0.61, respectively. Mixed-effects modeling identified chlorophyll, root vitality, soluble sugars, and photosynthesis as the primary determinants of stay-green in *E. sibiricus*. The TOPSIS model indicated that HB-15 maintained the highest fitting degree values in years 2–4. These values were 0.69, 0.62, and 0.71, respectively. Therefore, HB-15 was the most ideal stay-green germplasm. These findings provide a theoretical basis and elite parental materials for breeding new stay-green varieties of *E. sibiricus*.

## 1. Introduction

The Qinghai–Tibet Plateau has abundant grassland resources and diverse forage species, serving as a major base for livestock production in China [1]. However, due to the region’s unique conditions of high elevation, large diurnal temperature variation, and a short growing season [2], forage grasses commonly exhibit early senescence that “turns yellow at the onset of autumn”, which shortens the period of sustained supply of green herbage and sharply reduces nutritional quality [3,4]. This has become a key bottleneck limiting the efficient and sustainable development of grassland animal husbandry and the effectiveness of ecological restoration of degraded grasslands. Therefore, breeding new forage varieties with delayed senescence is an effective way to overcome this bottleneck.

Plant senescence represents a highly programmed process that is coordinately regulated by oxidative metabolism, hormone signaling, and gene expression. Chlorophyll degradation, photosynthetic capacity decline, and nutrient remobilization in leaves are the hallmark features of this process. The dynamic equilibrium among reactive oxygen species (ROS), abscisic acid (ABA), and cytokinin (CTK) within the plant governs the initiation, rate, and termination of senescence; any disturbance of this balance may either accelerate or delay senescence [5,6]. Stay-green is an important trait that delays the progression of senescence, manifested as the prolonged maintenance of leaf greenness and normal photosynthetic physiology during the late reproductive stage [7,8]. This trait is not only key to extending the duration of photosynthesis and increasing biomass, but also a fundamental basis for enhancing stress tolerance and stabilizing yield [9,10]. Extensive studies on stay-green plants have been reported, mainly in perennial ryegrass [7], maize [11], sorghum [12,13], wheat [14,15], and rice [16,17]. These studies showed that stay-green is a beneficial trait that increases yield, strengthens stress resistance, and delays senescence; it is genetically regulated, stably inherited, and its genetic mechanisms and physiological basis have been preliminarily elucidated [7,11,17]. Accordingly, undertaking the collection and research of perennial stay-green grass germplasm is of considerable theoretical and practical significance for improving the efficiency of ecological restoration and preserving biodiversity on the Qinghai–Tibet Plateau, enhancing forage production in native grasslands, prolonging the availability of green fodder, and reconciling forage supply–demand imbalances during the autumn and winter seasons.

*Elymus sibiricus* is a perennial, high-quality, cool-season forage grass belonging to the Poaceae family. It has a strong adaptation and high feeding value. It plays an essential role in livestock production and ecological restoration of degraded grasslands on the Qinghai–Tibet Plateau [18,19]. Current studies on *E. sibiricus* have focused on phenotypic traits and some physiological indices. Most studies have emphasized agronomic traits, forage yield, nutritional quality, and stress resistance [20,21,22,23,24]. A systematic analysis of the coordinated changes in phenotype and physiology during senescence in stay-green germplasm is lacking. A comprehensive evaluation system for the accurate selection of superior stay-green germplasm is also lacking.

Based on this, we used six preliminarily selected stay-green *E. sibiricus* germplasm lines as the study subjects and the non-stay-green cultivar *E. sibiricus* cv. Qingmu No. 2 was used as the control. A three-year fixed-site observational trial was conducted in a typical ecological zone of the Qinghai–Tibet Plateau (Haiyan County, Qinghai Province). The objectives were: (1) to elucidate dynamic changes in phenotypic and physiological traits during the critical period of senescence in stay-green *E. sibiricus*; (2) to reveal differences in stay-green capacity among germplasm lines and identify key influencing factors; and (3) by employing the TOPSIS model, to achieve the complete retention of indicator information, compatibility with diverse trait categories, and objective quantification, thereby allowing precise identification and selection of the best stay-green *E*. *sibiricus* germplasm resources that integrate stay-green traits with superior agronomic performance. These studies provide elite parental materials and a theoretical basis for breeding new stay-green *E. sibiricus* cultivars.

## 2. Results

### 2.1. Morphological and Structural Traits of E. sibiricus and Senescence Dynamics

**Figure 1:**
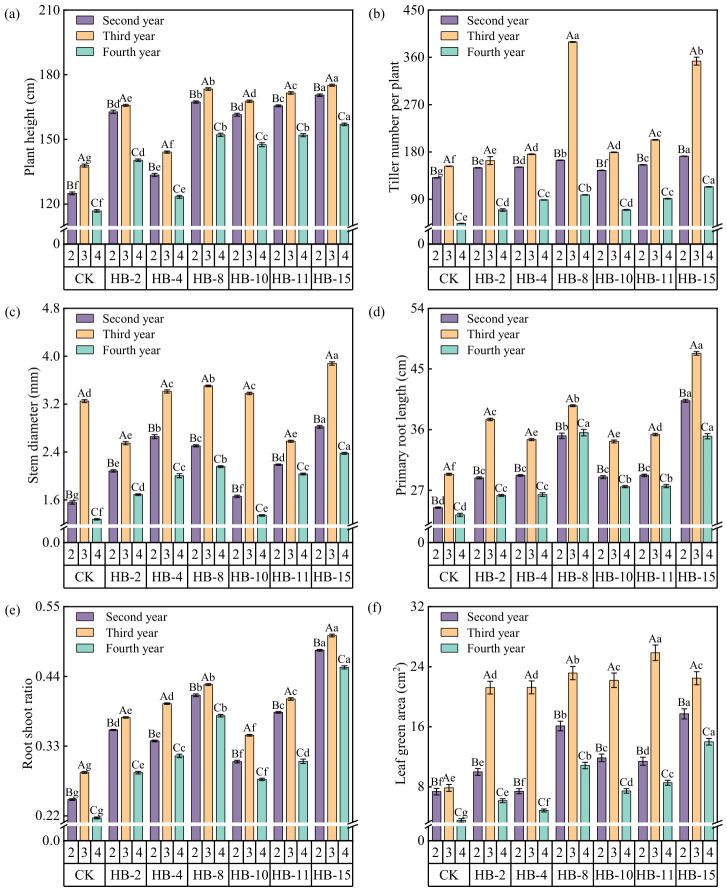

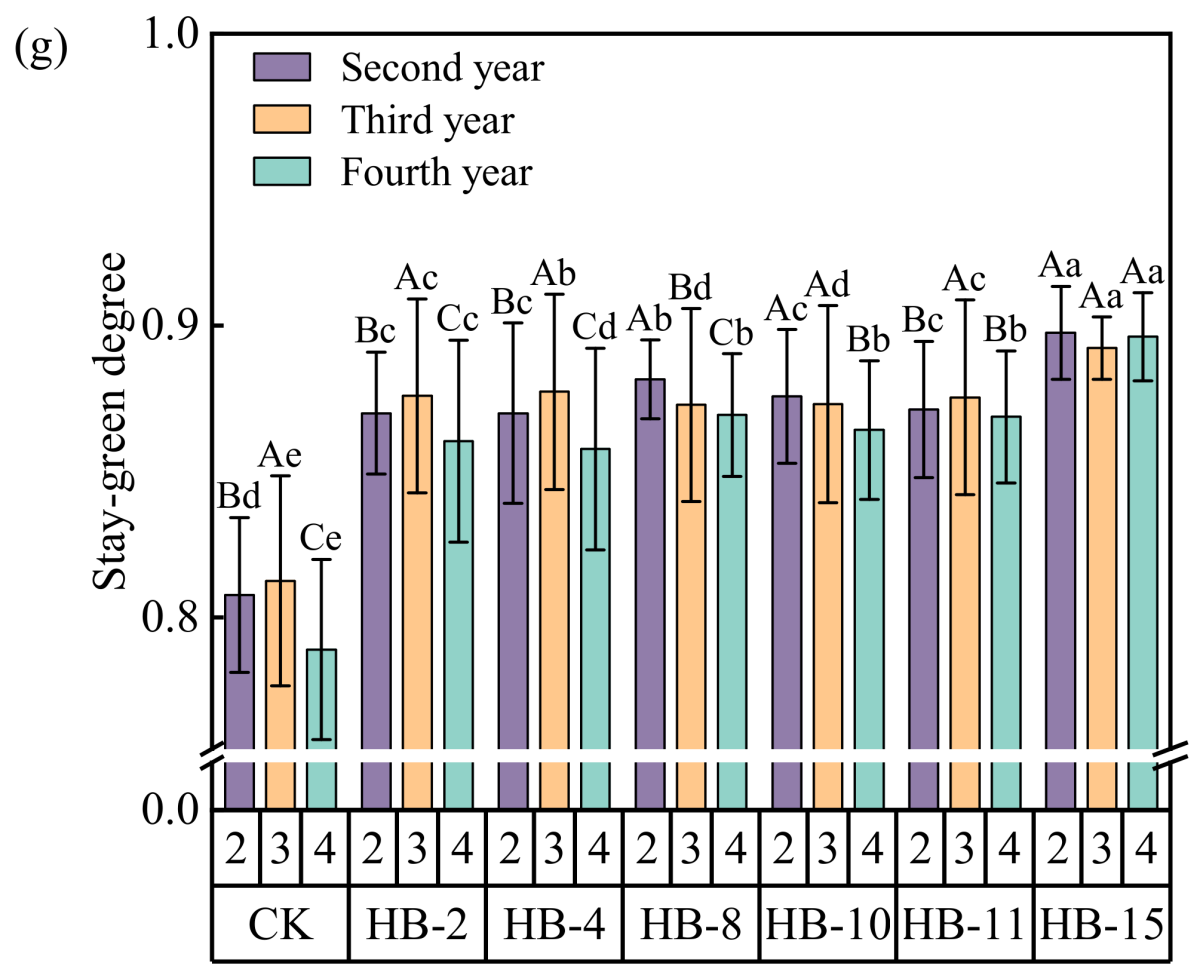
Morphological and structural characteristics of *E. sibiricus*. Lowercase letters indicate significant differences among *E. sibiricus* accessions within the same year, whereas uppercase letters indicate significant differences for the same accession across different years. The error above the column chart represents the standard error. The same convention applies throughout. (**a**) Plant height; (**b**) Tiller number per plant; (**c**) Stem diameter; (**d**) Primary root length; (**e**) Root-shoot ratio; (**f**) Leaf green area; (**g**) Stay-green degree.

The phenotypic traits of *E. sibiricus* germplasm differed significantly among planting years (Figure 1). Within the same germplasm, plant height, tiller number, stem diameter, primary root length, root shoot ratio, green leaf area, and stay-green degree were highest in the third year, intermediate in the second year, and lowest in the fourth year. The differences among the planting years were significant (*p* < 0.05).

From the second to fourth years, stay-green germplasm showed significantly superior plant height, tiller number, stem diameter, primary root length, root shoot ratio, green leaf area, and stay-green degree compared with non-stay-green germplasm (*p* < 0.05). In the second year, HB-15 had the highest plant height, the largest tiller number per plant, the greatest stem diameter, the longest primary root, the largest root shoot ratio, the largest green leaf area, and the best stay-green degree (*p* < 0.05). Values were 170.52 cm, 171.96 tillers per plant, 2.82 mm, 40.28 cm, 0.48, 17.73 cm^2^, and 0.89, respectively (Figure 1a–g). In the third year, HB-15 exhibited the highest plant height, stem diameter, primary root length, root shoot ratio, and stay-green degree, with values of 175.14 cm, 3.88 mm, 47.31 cm, 0.51, and 0.89. HB-8 had the largest tiller number per plant, with 389.21 tillers per plant. HB-11 had the largest green leaf area, at 25.86 cm^2^. In the fourth year, HB-15 again showed the highest plant height, the largest tiller number per plant, the greatest stem diameter, the longest primary root, the largest root shoot ratio, the largest green leaf area, and the best stay-green degree (*p* < 0.05). Values were 156.98 cm, 113.86 tillers per plant, 2.38 mm, 35.02 cm, 0.45, 14.01 cm^2^, and 0.90.

**Figure 2:**
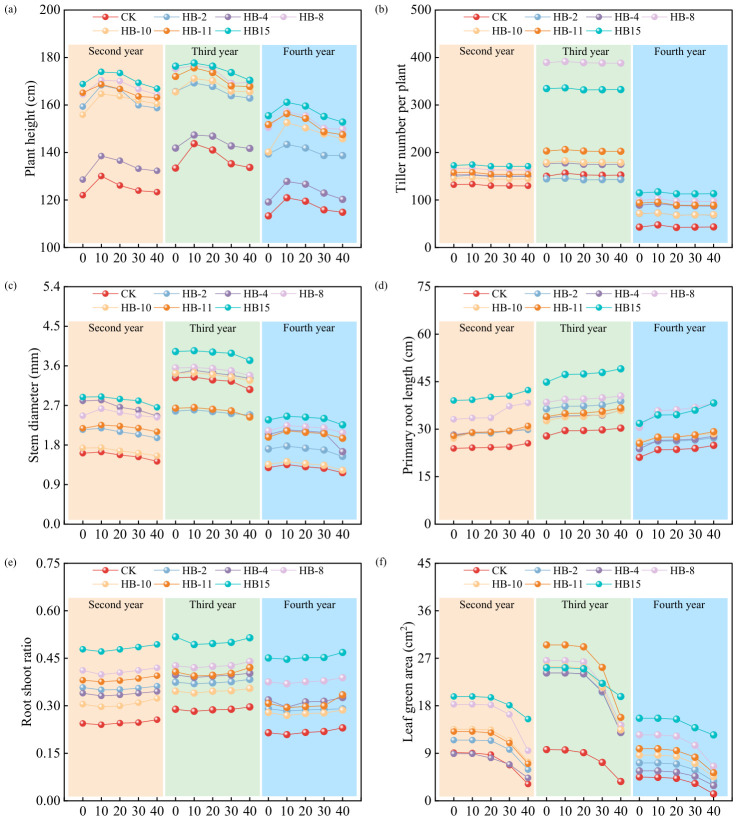

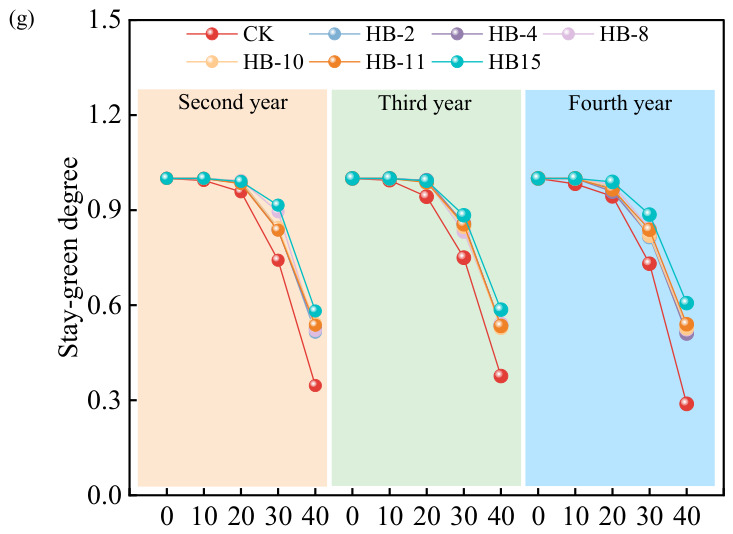
Phenotypic dynamic changes in *E. sibiricus*. “0–40” denotes the time points of sampling and observation. The same convention applies throughout. (**a**) Plant height; (**b**) Tiller number per plant; (**c**) Stem diameter; (**d**) Primary root length; (**e**) Root-shoot ratio; (**f**) Leaf green area; (**g**) Stay-green degree.

As senescence progressed after heading, stay-green *E. sibiricus* consistently showed superior plant height, tiller number, stem diameter, primary root length, root shoot ratio, green leaf area, and stay-green degree compared with non-stay-green plant materials (Figure 2). From the second to fourth years, plant height after heading first increased and then decreased; it reached a maximum at 10 d and then declined, and it began to stabilize at 30 d (Figure 2a). From the second to fourth years, tiller number per plant after heading changed slightly; it first increased and then decreased, reached a maximum at 10 d, then declined, and began to stabilize at 20 d (Figure 2b). In the second and third years, stem diameter showed an overall decreasing trend; in the fourth year, it first increased and then decreased (Figure 2c). From the second to fourth years, primary root length showed a gradual increase; growth slowed at 10–20 d (Figure 2d). From the second to fourth years, the root shoot ratio after heading first decreased and then increased during aging; it was minimal at 10 d and then gradually increased (Figure 2e). From the second to fourth years, in stay-green *E. sibiricus* plants, green leaf area began to decrease at 20 d, with HB-15 decreasing more slowly; in non-stay-green plants, green leaf area began to decrease at 10 d and decreased rapidly starting at 30 d (Figure 2f). From the second to fourth years, in stay-green plants, stay-green degree began to decline at 20 d and declined rapidly starting at 30 d. In the second and third years, in non-stay-green plants, stay-green degree began to decline at 10 d and declined rapidly starting at 20 d; in the fourth year, stay-green degree declined after heading and declined rapidly at 20 d (Figure 2g). These results indicated that stay-green *E. sibiricus* senesced more slowly than non-stay-green plant materials. In the later stage of plant senescence, it maintained a larger green leaf area and higher stay-green degree, which laid a foundation for sustaining photosynthesis during late senescence.

### 2.2. Photosynthetic Characteristics and Senescence Patterns of E. sibiricus

**Figure 3:**
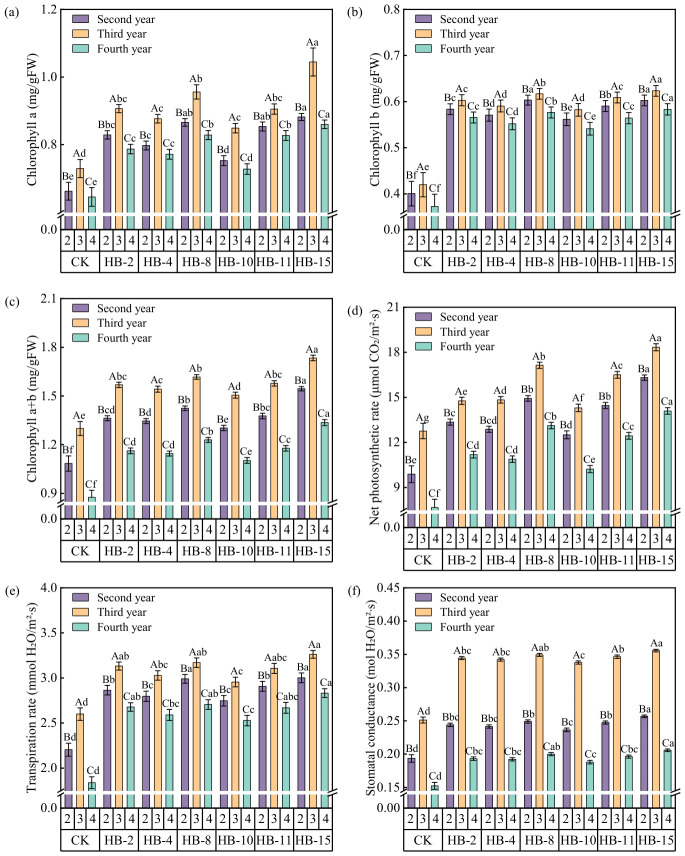

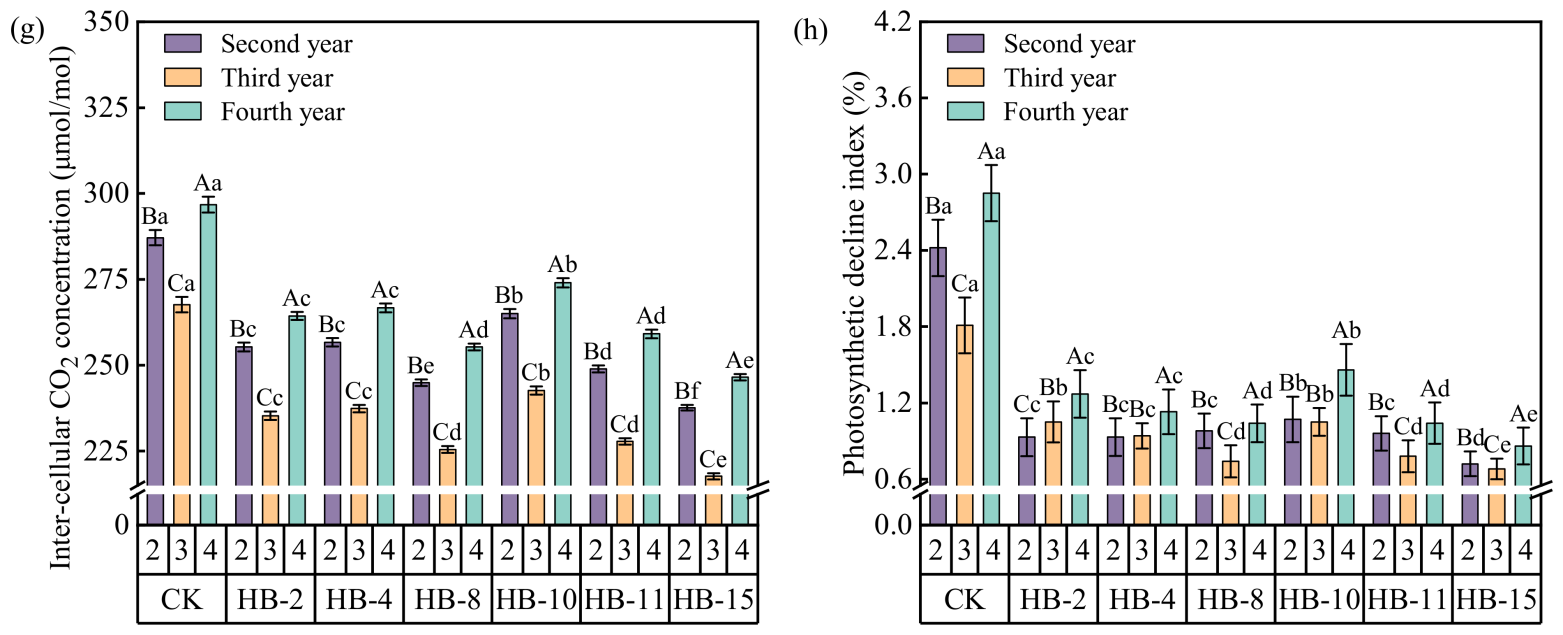
Photosynthetic characteristics of *E. sibiricus.* Lowercase letters indicate significant differences among *E. sibiricus* accessions within the same year, whereas uppercase letters indicate significant differences for the same accession across different years. The error above the column chart represents the standard error. (**a**) Chlorophyll a; (**b**) Chlorophyll b; (**c**) Chlorophyll a + b; (**d**) Net photosynthetic rate; (**e**) Transpiration rate; (**f**) Stomatal conductance; (**g**) Inter-cellular CO_2_ concentration; (**h**) Photosynthetic decline index.

The photosynthetic characteristics represented significant differences among *E. sibiricus* germplasm planting years (Figure 3). Within the same germplasm, chlorophyll a, chlorophyll b, chlorophyll a + b, net photosynthetic rate, transpiration rate, stomatal conductance, inter-cellular CO_2_ concentration, and the photosynthetic decline index showed significant differences among planting years (*p* < 0.05) and stay-green plants performed better than non-stay-green plants. In the second year, HB-15 had the highest chlorophyll a + b content, net photosynthetic rate, and stomatal conductance, with 1.54 mg/gFW, 16.31 μmol CO_2_/m^2^·s, and 0.26 mol H_2_O/m^2^·s, respectively (Figure 3c,d,f). HB-15 and HB-8 had the highest chlorophyll b content and transpiration rate, with 0.60 mg/gFW and 3.00 mmol H_2_O/m^2^·s, respectively (Figure 3b,e). HB-15, HB-11, and HB-8 had the highest chlorophyll a content, at 0.85–0.88 mg/gFW (Figure 3a). Inter-cellular CO_2_ concentration and the photosynthetic decline index were higher in non-stay-green plants than in stay-green plants, with HB-15 being the lowest (Figure 3g,h). In the third year, HB-15 had the highest chlorophyll a, chlorophyll b, chlorophyll a + b, and net photosynthetic rate, with 1.04 mg/gFW, 0.62 mg/gFW, 1.73 mg/gFW, and 18.33 μmol CO_2_/m^2^·s, respectively (Figure 3a–d). HB-15 and HB-8 had the highest transpiration rate and stomatal conductance (Figure 3e,f). Inter-cellular CO_2_ concentration and the photosynthetic decline index were higher in non-stay-green plants than in stay-green plants, with HB-15 being the lowest (Figure 3g,h). In the fourth year, HB-15 had the highest chlorophyll a, chlorophyll b, chlorophyll a + b, and net photosynthetic rate, with 0.86 mg/gFW, 0.58 mg/gFW, 1.33 mg/gFW, and 14.09 μmol CO_2_/m^2^·s, respectively (Figure 3a–d). HB-15 and HB-8 had the highest transpiration rate and stomatal conductance (Figure 3e,f). Inter-cellular CO_2_ concentration and the photosynthetic decline index were higher in non-stay-green plants than in stay-green plants, with HB-15 being the lowest (Figure 3g,h).

**Figure 4:**
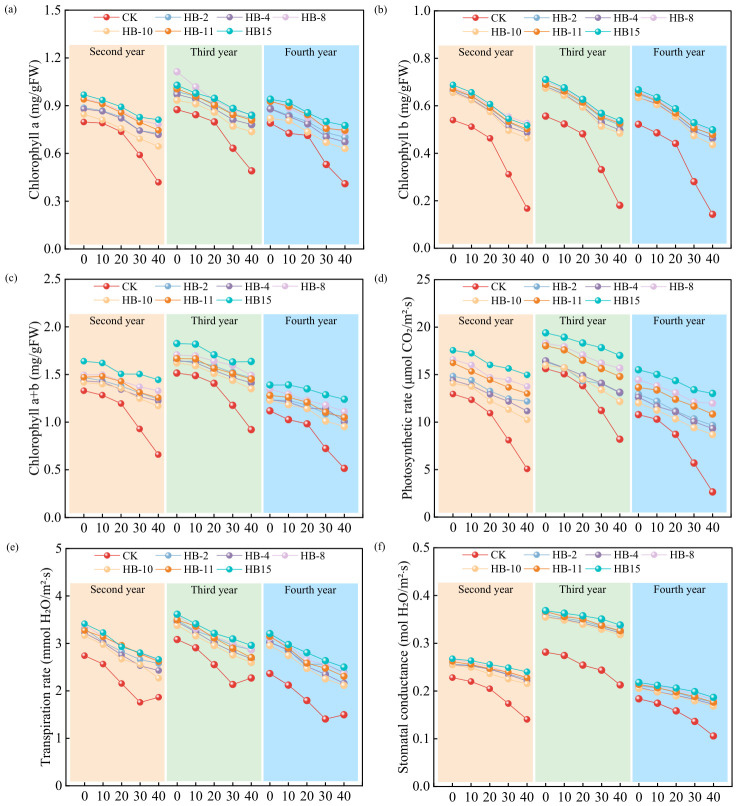

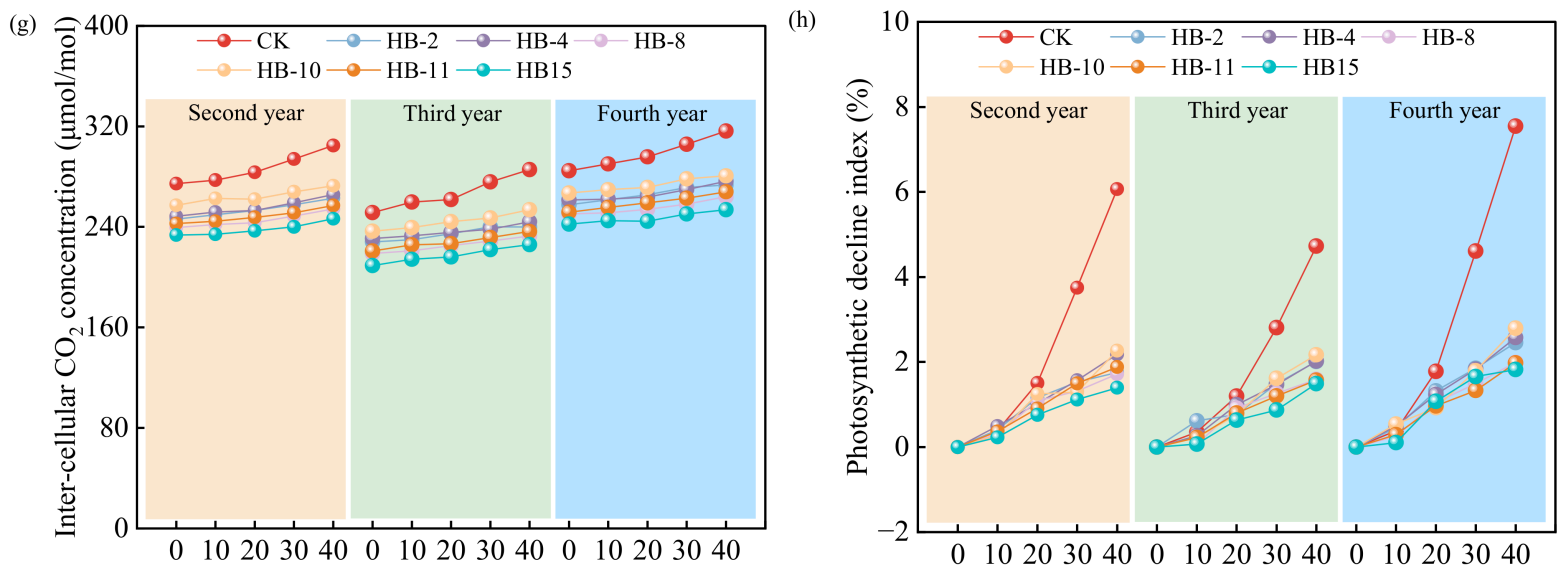
Photosynthetic dynamic changes in *E. sibiricus.* (**a**) Chlorophyll a; (**b**) Chlorophyll b; (**c**) Chlorophyll a + b; (**d**) Net photosynthetic rate; (**e**) Transpiration rate; (**f**) Stomatal conductance; (**g**) Inter-cellular CO_2_ concentration; (**h**) Photosynthetic decline index.

As senescence progressed after heading, stay-green *E. sibiricus* showed superior chlorophyll a, chlorophyll b, total chlorophyll (a + b), net photosynthetic rate, transpiration rate, stomatal conductance, inter-cellular CO_2_ concentration, and photosynthetic decline index compared with non-stay-green plant materials (Figure 4).

From the second to fourth years, chlorophyll a content, chlorophyll b content, chlorophyll a + b content, net photosynthetic rate, and stomatal conductance in the flag leaves of the tested *E. sibiricus* showed continuous declines. Starting at 20 d after heading, non-stay-green plants showed rapid declines in chlorophyll a, chlorophyll b, chlorophyll a + b, net photosynthetic rate, and stomatal conductance, whereas stay-green plants declined more slowly, with HB-15 performing better (Figure 4a–d,f). In the later stage of plant senescence, they still maintained higher chlorophyll content and photosynthetic capacity. The transpiration rate in stay-green plants showed an overall decreasing trend, whereas non-stay-green plants first decreased and then increased; it was lowest at 30 d after heading, gradually increasing at 30–40 d (Figure 4e). Inter-cellular CO_2_ concentration and the photosynthetic decline index showed gradual increases. Non-stay-green plants increased faster and began to rise rapidly at 20 d, whereas stay-green plants changed more slowly, with HB-15 performing better. In the later stage of plant senescence, they still maintained higher photosynthetic conversion capacity and stronger anti-senescence capacity (Figure 4g,h). These results indicated that the flag leaves of stay-green germplasm senesced more slowly. In the later stage of plant senescence, they still maintained higher chlorophyll content and photosynthetic capacity, which greatly improved abiotic carbon–nitrogen cycling and energy flow efficiency.

### 2.3. Physiological Characteristics and Senescence Patterns of E. sibiricus

**Figure 5:**
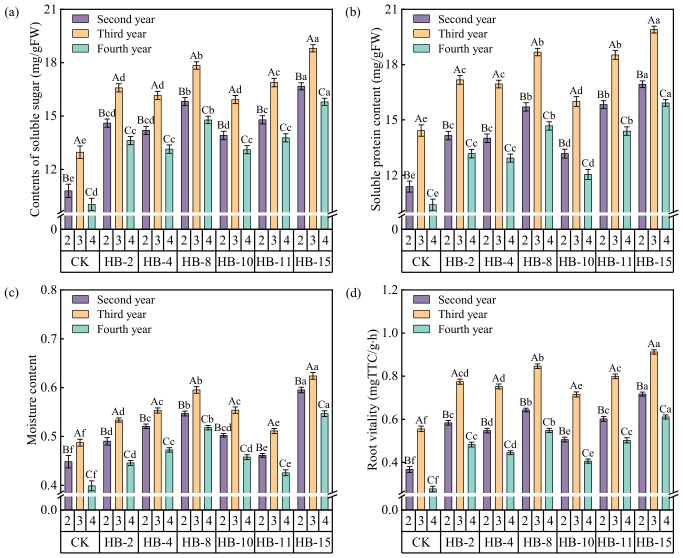
Physiological characteristics of *E. sibiricus.* Lowercase letters indicate significant differences among *E. sibiricus* accessions within the same year, whereas uppercase letters indicate significant differences for the same accession across different years. The error above the column chart represents the standard error. (**a**) contents of soluble sugar; (**b**) Soluble protein content; (**c**) Moisture content; (**d**) Root vitality.

**Figure 6:**
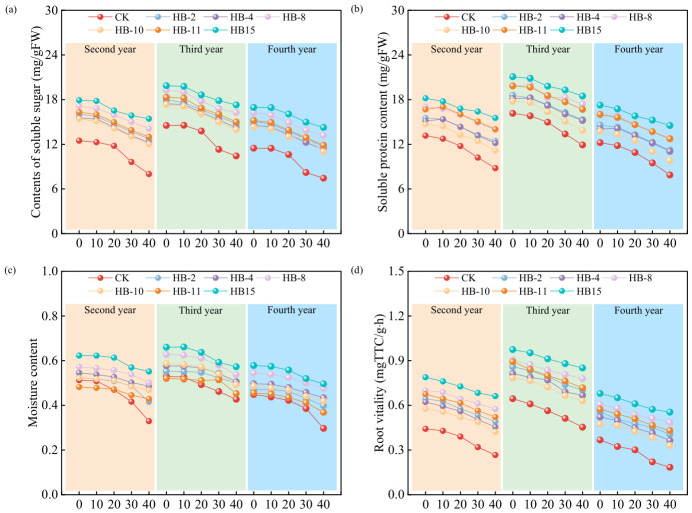
Physiological dynamic changes in *E. sibiricus.* (**a**) contents of soluble sugar; (**b**) Soluble protein content; (**c**) Moisture content; (**d**) Root vitality.

The physiological characteristics of *E. sibiricus* germplasm displayed significant differences among planting years. Within the same germplasm, soluble sugar content, soluble protein content, moisture content, and root activity had significant differences. They were highest in the third year, intermediate in the second year, and lowest in the fourth year; stay-green *E. sibiricus* were significantly superior to non-stay-green *E. sibiricus* (*p* < 0.05) (Figure 5). HB-15 exhibited significantly the highest soluble sugar content, soluble protein content, moisture content, and root activity (*p* < 0.05), with 18.81 ± 0.21–15.79 ± 0.24 mg/g FW, 19.90 ± 0.19–15.92 ± 0.18 mg/g FW, 0.62 ± 0.0069–0.55 ± 0.0058, and 0.91 ± 0.010–0.61 ± 0.0093 mg TTC/g·h, respectively (Figure 6a–d).

As senescence progressed after heading, *E. sibiricus* germplasm showed decreasing trends in soluble sugar content, soluble protein content, moisture content, and root activity across planting years, with stay-green materials declining more slowly than non-stay-green materials (Figure 6). In the second year, in non-stay-green materials, soluble sugar content dropped rapidly at 20 d after heading, while soluble protein content, moisture content, and root activity began to decline rapidly from 10 d after heading. In the late stage of plant senescence, stay-green materials still maintained higher levels of soluble sugars, soluble proteins, moisture content, and root activity. In the third year, in non-stay-green materials, soluble sugar and soluble protein content decreased rapidly at 20 d after heading; moisture content declined rapidly during the late stage of plant senescence. In the fourth year, in non-stay-green materials, soluble sugar content, soluble protein content, and moisture content began to decrease rapidly at 20 d after heading. In stay-green materials, the decline rates of soluble sugar content, soluble protein content, moisture content, and root activity were slower. These results indicated that, compared with non-stay-green materials, stay-green *E. sibiricus* maintained higher levels of osmotic regulatory substances and stronger root activity during the late stage of plant senescence, and supported anti-senescence, stress resistance, and the normal translocation of nutrients.

### 2.4. Correlation Analysis Between Stay-Green Phenotypes and Physiological Traits in E. sibiricus

**Figure 7:**
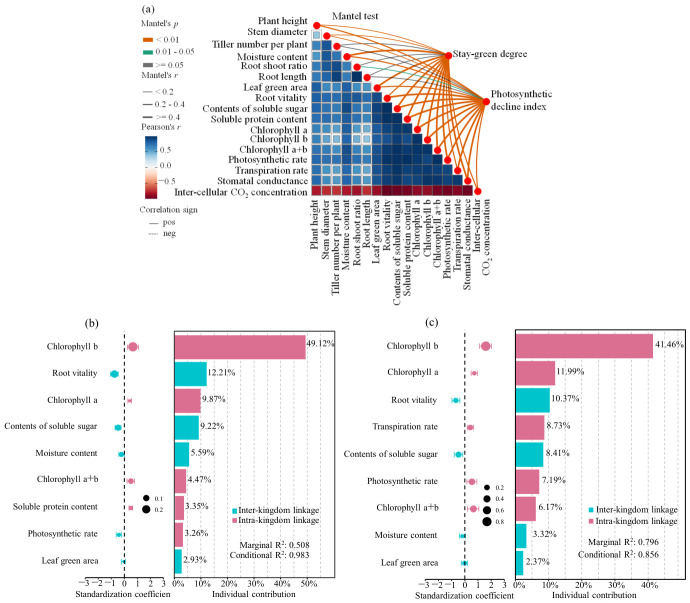
Correlation analysis between phenotypic and physiological traits in *E. sibiricus* based on the Mantel test and mixed–effects models. In (**a**), the thickness of the connecting lines indicates the strength of the correlation. (**b**) presents the analysis of the relationships between stay-green and its influencing factors, while (**c**) presents the correlation analysis between the photosynthetic decline index and its influencing factors. (**a**) Mantel test correlation analysis; (**b**) Analysis of the mixed effects of stay–green; (**c**) Mixed–effects analysis of photosynthetic decline index.

The Mantel test analysis of correlations among stay-green degree, photosynthetic decline index, and phenotypic, photosynthetic, and physiological traits showed significant differences (Figure 7a). The stay-green degree was highly significantly correlated with plant height, stem diameter, moisture content, green leaf area, root activity, soluble sugar content, soluble protein content, chlorophyll a, chlorophyll b, chlorophyll a + b, net photosynthetic rate, transpiration rate, stomatal conductance, and inter-cellular CO_2_ concentration (*p* < 0.01). The photosynthetic decline index was highly significantly correlated with plant height (*p* < 0.01), stem diameter, moisture content, green leaf area, root activity, soluble sugar content, soluble protein content, chlorophyll a, chlorophyll b, chlorophyll a + b, net photosynthetic rate, transpiration rate, stomatal conductance, and inter-cellular CO_2_ concentration, and was significantly correlated with the root shoot ratio (*p* < 0.05).

Based on the Mantel test with respect to derived correlation analysis, we selected factors that significantly affect stay-green degree and the photosynthetic decline index, and further built mixed-effects models to quantify the primary drivers of stay-green in *E. sibiricus*. The model for stay-green degree fitted well (marginal R^2^ = 0.508; conditional R^2^ = 0.983), identifying chlorophyll content, root activity, moisture content, soluble sugar content, soluble protein content, and net photosynthetic rate as key influencing factors. Among these, chlorophyll b, root activity, chlorophyll a, and soluble sugars jointly explained approximately 80.42% (Figure 7b).

The mixed-effects model for the photosynthetic decline index also showed good fit (marginal R^2^ = 0.796; conditional R^2^ = 0.856). Key factors include chlorophyll content, root activity, net photosynthetic rate, transpiration rate, soluble sugar content, and moisture content, with chlorophyll b, chlorophyll a, root activity, transpiration rate, and soluble sugar content jointly explaining approximately 80.96% (Figure 7c). Collectively, chlorophyll metrics, root activity, soluble sugars, and photosynthetic performance emerged as the principal determinants of the stay-green trait in *E. sibiricus*.

### 2.5. Variation Analysis and Comprehensive Evaluation of Stay-Green Phenotypes and Photosynthetic and Physiological Characteristics in E. sibiricus

#### 2.5.1. Variation Analysis of Phenotypes and Photosynthetic and Physiological Characteristics in *E. sibiricus*

The variation in phenotypic, photosynthetic, and physiological characteristics of stay-green *E. sibiricus* germplasm was analyzed (Table 1). The results showed that the coefficient of variation for tiller number per plant was the largest (0.54). The coefficient of variation for green leaf area was (0.52). The coefficient of variation for the photosynthetic decline index was (0.49). The coefficients of variation for stem diameter, root shoot ratio, root activity, and stomatal conductance were relatively large, at (0.31), (0.21), (0.27), and (0.26), respectively. These findings indicated that leaf stay-green did not equal normal function. To avoid selecting “green but functionally ineffective” plants, it was crucial and necessary to construct a comprehensive evaluation system of key factors affecting stay-green in *E. sibiricus*.

#### 2.5.2. Screening and Comprehensive Evaluation of Stay-Green *E. sibiricus* Germplasm

Based on the variation analysis of all factors, to further screen *E. sibiricus* germplasm with superior stay-green performance, we used the TOPSIS model to comprehensively evaluate all tested germplasm resources across phenotypic traits (plant height, tiller number per plant, stem diameter, primary root length, root shoot ratio, green leaf area, stay-green degree), photosynthetic traits (chlorophyll a, chlorophyll b, total chlorophyll a + b, net photosynthetic rate, transpiration rate, stomatal conductance, inter-cellular CO_2_ concentration, photosynthetic decline index), and physiological traits (soluble sugars, soluble proteins, moisture content, root activity).

In the second year, plant height was 0.055; stem diameter, 0.074; tiller number per plant, 0.052; moisture content, 0.097; root shoot ratio, 0.070; primary root length, 0.087; green leaf area, 0.121; stay-green degree, 0.047; root activity, 0.056; soluble sugar content, 0.052; soluble protein content, 0.063; chlorophyll a, 0.056; chlorophyll b, 0.046; chlorophyll a + b, 0.052; net photosynthetic rate, 0.059; transpiration rate, 0.047; stomatal conductance, 0.047; inter-cellular CO_2_ concentration, 0.053; and photosynthetic decline index, 0.047. In the third year, plant height was 0.056; stem diameter, 0.074; tiller number per plant, 0.145; moisture content, 0.097; root shoot ratio, 0.074; primary root length, 0.086; green leaf area, 0.050; stay-green degree, 0.050; root activity, 0.060; soluble sugar content, 0.057; soluble protein content, 0.069; chlorophyll a, 0.062; chlorophyll b, 0.049; chlorophyll a + b, 0.058; net photosynthetic rate, 0.077; transpiration rate, 0.055; stomatal conductance, 0.049; inter-cellular CO_2_ concentration, 0.056; and photosynthetic decline index, 0.051. In the fourth year, plant height was 0.059; stem diameter, 0.076; tiller number per plant, 0.051; moisture content, 0.097; root shoot ratio, 0.077; primary root length, 0.090; green leaf area, 0.061; stay-green degree, 0.051; root activity, 0.061; soluble sugar content, 0.056; soluble protein content, 0.069; chlorophyll a, 0.060; chlorophyll b, 0.049; chlorophyll a + b, 0.057; net photosynthetic rate, 0.066; transpiration rate, 0.051; stomatal conductance, 0.049; inter-cellular CO_2_ concentration, 0.056; and photosynthetic decline index, 0.050. In terms of combined weights, plant height was 0.056; stem diameter, 0.107; tiller number per plant, 0.090; moisture content, 0.097; root shoot ratio, 0.077; primary root length, 0.090; green leaf area, 0.061; stay-green degree, 0.051; root activity, 0.061; soluble sugar content, 0.056; soluble protein content, 0.069; chlorophyll a, 0.060; chlorophyll b, 0.049; chlorophyll a + b, 0.057; net photosynthetic rate, 0.066; transpiration rate, 0.051; stomatal conductance, 0.049; inter-cellular CO_2_ concentration, 0.056; and photosynthetic decline index, 0.050 (Table 2).

**Figure 8:**
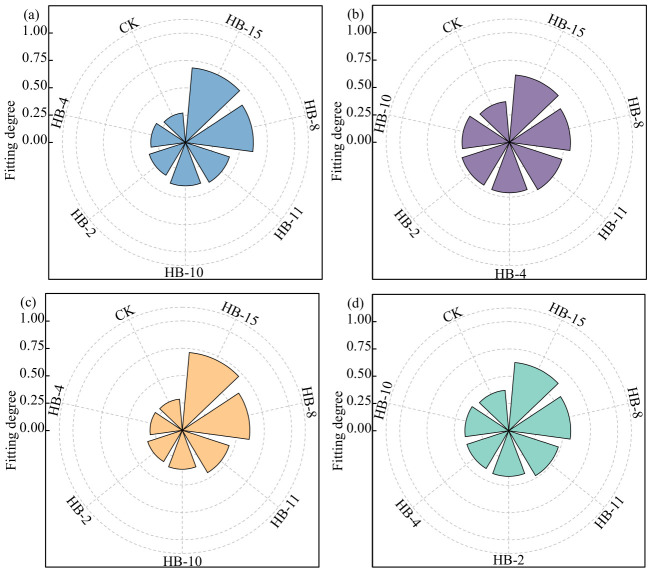
Comprehensive evaluation of stay-green *E. sibiricus* germplasm resources based on the TOPSIS model. (**a**) Comprehensive evaluation result at the second year; (**b**) Comprehensive evaluation result at the third year; (**c**) Comprehensive evaluation result at the fourth year; (**d**) Overall comprehensive evaluation result.

The comprehensive evaluation based on the TOPSIS model showed that, from the second to fourth years, HB-15 performed best overall and CK performed worst. In the second year, the degree of fit ranked from high to low, as follows: HB-15, HB-8, HB-11, HB-10, HB-2, HB-4, and CK (Figure 8a). In the third year, the ranking was HB-15, HB-8, HB-11, HB-4, HB-2, HB-10, and CK (Figure 8b). In the fourth year, the ranking was HB-15, HB-8, HB-11, HB-10, HB-2, HB-4, and CK (Figure 8c). The aggregated comprehensive fit ranked as follows: HB-15, HB-8, HB-11, HB-2, HB-4, HB-10, and CK, with HB-15 highest (0.63) and CK lowest (0.37) (Figure 8d). Taken together, HB-15 exhibited the best overall performance and can be considered the most suitable parental material for breeding new stay-green *E. sibiricus* varieties.

## 3. Discussion

Stay-green is a beneficial trait that delays leaf senescence in the late reproductive stage, maintains photosynthetic function, and enhances stress tolerance and yield stability [25]. Phenotypic traits are direct indicators of plant growth and environmental adaptation, and their senescence rates directly reflect stay-green capacity [14]. We found that, in the second to fourth year, stay-green *E. sibiricus* had significantly higher plant height, stem diameter, tiller number per plant, green leaf area, and stay-green degree than non-stay-green materials (*p* < 0.05), with markedly reduced senescence rates. Among the tested stay-green *E. sibiricus* germplasms, HB-15 exhibited the best overall performance. For years 2–4 years, all indices—including plant height, tiller number per plant, green leaf area, and stay-green degree—followed the pattern of peaking in the third year, followed by the second year, and reaching the minimum in the fourth year. This trend was consistent with the growth pattern of all tested *E. sibiricus* germplasms and highly aligned with the resource allocation strategy of alpine plants on the Qinghai–Tibet Plateau [26,27]. In the third year, plants are in vigorous growth, with sufficient accumulation of photosynthates; stem toughness, root extension capacity, and tillering ability are optimal. Healthy stems and roots underpin efficient transport of water, nutrients, and photosynthates, laying a material foundation for delayed senescence [28,29]. In the fourth year, long-term environmental stress and nutrient depletion cause a slight decline in phenotypic indices, yet the stay-green germplasm still shows significantly smaller declines than non-stay-green materials, indicating genetic stability. This finding addressed the limitation of “single-age evaluation” in forage resources of alpine grassland regions and provided an important basis for germplasm introduction and variety breeding in alpine grassland regions.

Sustained stability of photosynthetic function is the core hallmark of functional stay-green [25,30]. We found that stay-green *E. sibiricus* had significantly higher chlorophyll content (chlorophyll a, chlorophyll b, chlorophyll a + b), net photosynthetic rate (Pn), and stomatal conductance (Gs) than non-stay-green germplasm materials, with slower declines (Figure 5 and Figure 6). HB-15 was the most outstanding germplasm: in the third year, chlorophyll a was 1.04 mg/g FW, chlorophyll b was 0.62 mg/g FW, and chlorophyll a + b was 1.73 mg/g FW; the net photosynthetic rate reached 18.33 μmol CO_2_/m^2^ s, significantly higher than non-stay-green germplasm materials. The photosynthetic decline index was only 0.0068, significantly lower than non-stay-green germplasm materials, indicating a markedly slower decline of photosynthetic function. These results fully supported the “functional stay-green” theory proposed by Thomas et al. [25]. They indicated that stay-green in *E. sibiricus* was not merely chlorophyll “retention,” but maintenance of photosynthetic efficiency via dual mechanisms: first, suppression of chlorophyll degradation pathways reduces enzymatic breakdown of chlorophyll a and chlorophyll b, allowing leaves to retain high pigment levels in late senescence; second, structural stability of photosystem II (PSII) is maintained, and dynamic regulation of stomatal conductance ensured CO_2_ supply and transporting of photosynthates, preventing a “green but non-functional” stay-green. However, previous experiments did not analyze chlorophyll fluorescence parameters in stay-green *E. sibiricus*. Therefore, determining whether *E. sibiricus* exhibits functional stay-green solely based on chlorophyll content changes and photosynthetic parameters has certain limitations. Thus, exploring the relationship between chlorophyll fluorescence parameters and stay-green traits in *E. sibiricus* is particularly important in future research. Furthermore, our study further revealed an adaptation mechanism of *E. sibiricus* under alpine grassland conditions: stay-green materials mitigated the rise in the rate of inter-cellular CO_2_ concentration (Ci) in late senescence (the increased rate with respect to Ci was significantly lower than in non-stay-green materials). This is because, during natural leaf senescence, the photosynthetic apparatus is gradually damaged. The photosynthetic carbon assimilation capacity also declines continuously. Consequently, the plant’s efficiency in fixing and utilizing inter-cellular CO_2_ (Ci) decreases. CO_2_ tends to accumulate in mesophyll inter-cellular spaces. This ultimately results in a substantial increase in Ci. An abnormal rise in Ci is usually accompanied by a significant decrease in photosynthetic rate. It is a typical physiological indicator of photosynthetic system inhibition. Compared with non-stay-green materials, stay-green *E. sibiricus* delays the increase in Ci. This indicates that the carbon fixation process is less disturbed during its senescence stage. It also demonstrates a stronger capacity to reduce photosynthetic inhibition. This is an adaptive strategy to highlight intensity and low temperature environments on the plateau. It is also a key distinction between stay-green and non-stay-green *E. sibiricus* materials [31], and it provides a new perspective for elucidating the photosynthetic regulatory network of stay-green in alpine forages.

**Figure 9:**
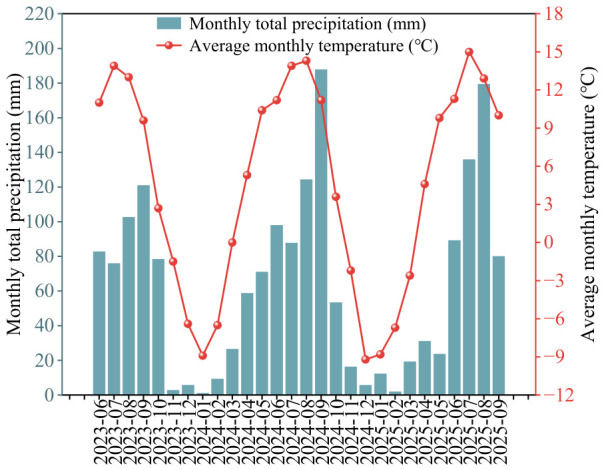
Distribution of monthly average temperature and precipitation at the experimental site from June 2023 to September 2025.

Physiological and metabolic indices (soluble sugars, soluble proteins, root activity) are core supports for stress tolerance and delayed senescence in plants [32,33]. We found that stay-green *E. sibiricus* maintained high levels of osmotic regulators and root activity during senescence. In the second to fourth years, HB-15 had soluble sugars of 15.79–18.81 mg/g FW, soluble proteins of 15.92–19.90 mg/g FW, and root activity of 0.61–0.91 mg TTC/g·h, which were 28.6–35.2%, 31.4–38.7%, and 42.3–51.8% higher than non-stay-green germplasm materials, respectively, with slower decline rates (Figure 7 and Figure 8). Soluble sugars and soluble proteins, as key osmotic regulators [34], not only maintained cellular osmotic pressure and mitigated oxidative damage to membranes during senescence but also supported chlorophyll synthesis and the activity of photosynthetic enzymes by providing energy and precursors [35,36]. Strong root activity enables sustained uptake of water and nutrients (N, P) in late senescence, underpinning the stability of photosynthetic function and aboveground phenotypes [37,38,39]. Mixed-effects modeling showed that chlorophyll b (explained variance 23.7%), root activity (19.5%), and soluble sugars (17.2%) were major factors affecting stay-green degree, with a cumulative explained variance of about 80.42% (Figure 9). These results indicated that a coordinated “chlorophyll-root-carbon metabolism” regulatory network was the key physiological basis of stay-green in *E. sibiricus*, providing a new framework for functional gene selection in subsequent molecular breeding of stay-green materials.

The TOPSIS model comprehensive analysis showed that HB-15 was the best-performing stay-green *E. sibiricus* germplasm over the study period. This was because HB-15 maintained superior phenotypic, photosynthetic, and physiological traits in the second to fourth year, with slower rates of decline and good genetic stability. In addition, HB-15 exhibited a pronounced functional stay-green phenotype, with a higher stay-green degree (0.89–0.90) and concurrent excellence in functional indices, such as net photosynthetic rate and root activity, thereby avoiding a “green but non-functional” state. The identification of elite stay-green *E. sibiricus* provided new breeding references and scientific solutions to address the issue of pastures “yellowing at the onset of autumn” in this region. However, this study was a fixed-site, single-plant cultivation experiment. This approach has generalizability limitations. It differs significantly from the clump cultivation typical of actual grassland production. This difference may affect the generalizability of stay-green performance. Therefore, this study lays the foundation for future experiments at different sites and with different planting densities.

## 4. Materials and Methods

### 4.1. Experimental Site Description

The experimental site was located at the National Forage Germplasm Nursery in Haiyan County, Qinghai Province, on the Qinghai–Tibet Plateau (100°52.848′ E, 36°59.36′ N) at an altitude of 3156 m. The climate is typical of a plateau and is continental. Air is thin. Solar radiation is strong and sunshine is abundant. The weather is highly variable. The site has two seasons: cold and warm. The diurnal temperature range was large. The mean annual temperature is 0.9 °C. The region experiences an extremely short frost-free period of approximately 30 days annually, with frost possible during any month due to high-altitude radiative cooling. The plant-growing season is approximately 120 days. Rainfall and heat occur in the same season in this region. The mean annual precipitation is approximately 375 mm, concentrated from July to September. From June 2023 to September 2025, the monthly mean air temperature and precipitation were 4.6 °C and 64.1 mm, respectively (Figure 9). The mean annual sunshine duration is approximately 2980 h. The mean annual evaporation is approximately 1400 mm. The main soil type is Calcic Kastanozem (chestnut–calcareous soil). The soil organic matter, total nitrogen, total phosphorus, and available phosphorus contents were 32.5 g/kg, 1.6 g/kg, 1.4 g/kg, and 2.2 mg/kg, respectively. The soil pH was 8.4 [19].

### 4.2. Materials

The experimental materials were sourced from the Perennial Forage Germplasm Resource Nursery, Haibei Prefecture, maintained by the College of Animal Science and Veterinary Medicine, Qinghai University. In 2017, phenotypic screening identified *E. sibiricus* accessions retaining leaf and culm greenness during late ontogeny with maintained photosynthetic capacity, in contrast to the complete senescence of aboveground tissues in commercial perennial cultivars grown under identical conditions. This phenotypic divergence facilitated the acquisition of original materials with differential stay-green capacity, designated HB-1 through HB-18. Following hill-drop establishment in 2018 and triennial phenotypic evaluation (2018–2020), six accessions (HB-2, HB-4, HB-8, HB-10, HB-11, HB-15) were preliminarily selected, and they belong to stable accessions. Systematic characterization in 2022 employed *E. sibiricus* cv. Qingmu No. 2 as the control cultivar (Table 3 and Table S1).

### 4.3. Experimental Design

**Figure 10:**
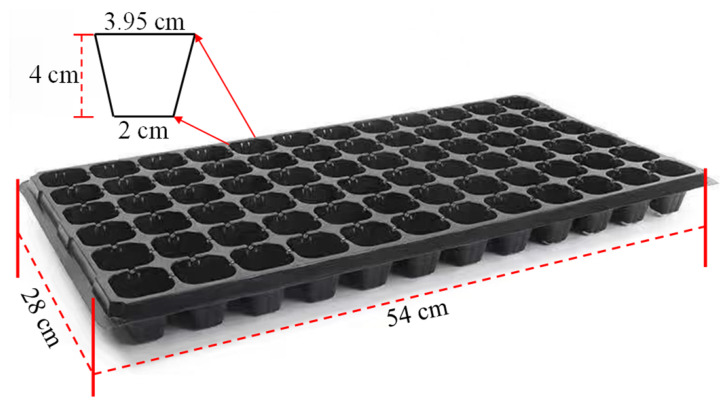
Seedling cell tray.

The experimental field was initiated by sowing in early June 2022. The experimental site was deeply plowed and leveled before sowing. Stones, roots, and other debris were removed from the soil. The experiment used fixed-site observations and sampling for three years. A completely randomized block design was used with six replicates. To maximize the production potential of *E. sibiricus*, plants were grown as single plants. Seeds were first germinated in seedling trays (tray size: 54 cm × 28 cm; 72-cell tray; top diameter, 2 cm; bottom, 3.95 cm; depth, 4 cm; single-cell volume, 28.8 mL). The trays were filled with mixed soil (field soil: nursery substrate = 1:1) (Figure 10). To improve emergence, the seeds were germinated in a greenhouse. After germination, the trays were moved to the field for acclimatization. Transplantation to the field was performed at the two-leaf, one-heart stage. Each plot measured 3 m × 5 m. The plant and row spacings were both 80 cm. A 1.5 m wide border row was established around the field. *E. sibiricus* cv. Qingmu No. 1 was used for the border row. The alleys between the plots were 1 m wide. Diammonium phosphate was used as the basal fertilizer at 75 kg/hm^2^. After seedling establishment, irrigation, fertilization, and grazing were prohibited in the study. Conventional field management was performed in the experimental field. In the first year (2022), weeding was performed thrice to ensure establishment and overwintering. In the second (2023), third (2024), and fourth (2025) years, inter-row cultivation and weeding were performed twice each year, after green-up and before stem elongation, along with plant disease, pest, and rodent control.

### 4.4. Sampling Methods and Index Measurement

Observations and measurements were initiated at heading in 2023 (second year), 2024 (third year), and 2025 (fourth year) for *E. sibiricus*. In each plot, 15 plants with similar growth and no visible pests or diseases were randomly selected. Sampling began at the heading stage. The sampling times were 0, 10, 20, 30, and 40 days after heading.

#### 4.4.1. Plant Height Measurement

Plant height is a key agronomic indicator of stress tolerance and production potential in crops. Measurement method: A steel tape was held vertically to the ground, and the distance from the plant base to the highest point of the plant under its natural growth posture was recorded. This value was defined as the plant height.

#### 4.4.2. Stem Diameter Measurement

Stem diameter is a core agronomic indicator that reflects stem toughness, lodging resistance, and the efficiency of water and nutrient transport, and is also a key metric of plant stress tolerance and production potential. Measurement method: Stem diameter was measured using a digital electronic caliper (accuracy 0.001 mm), with the measurement position standardized at the second internode for each plant.

#### 4.4.3. Moisture Content Measurement

Moisture content is a key indicator of plant water status, stress tolerance and nutritional quality. It is also a key indicator of physiological activities. Measurement method: In each plot, five single plants with uniform growth were randomly selected, avoiding edge effects. The plants were cut at ground level. Weeds were removed and weighed immediately to obtain the fresh mass. The *E. sibiricus* samples were placed in large paper envelopes and transported to the laboratory. First, viruses were inactivated at 105 °C for 30 min. The samples were then dried at 75 °C to a constant mass to obtain the dry mass. The moisture content was calculated as follows:

Moisture content = (Fresh mass − Dry mass)/(Dry mass)

#### 4.4.4. Tiller Number Measurement

The tiller number is a core agronomic indicator of clonal proliferation, biomass accumulation, and stress tolerance in grasses. It is also an important agronomic factor that affects forage yield and community stability. Measurement method: traditional per plant counting. The number of tillers per plant was counted. Tillers are defined as shoots that arise from the base and form normal stems.

#### 4.4.5. Root Shoot Ratio Measurement

The root shoot ratio is the ratio of the dry mass of the belowground parts to that of the aboveground parts. It is an important indicator of the allocation efficiency of photosynthates between belowground and aboveground organs of plants. It also reflects stress tolerance, nutrient uptake capacity, and the stability of the community. Measurement method: In each plot, five single plants with uniform growth were randomly excavated (excluding edge effects). The belowground and aboveground parts were separated at the crown, the roots were washed clean with water, and the aboveground and belowground materials were bagged separately in large envelopes and taken to the laboratory. Samples were first deactivated at 105 °C for 30 min, then oven-dried at 75 °C to a constant weight, and the dry mass was recorded.

Root shoot ratio = (Belowground dry mass)/(Aboveground dry mass)

#### 4.4.6. Primary Root Length Measurement

Primary root length is a core indicator of root extension capacity and the ability to absorb nutrients and water in *E. sibiricus* plants. Measurement method: The intact natural length of the single-plant root system was measured. A steel tape was used to record the distance from the plant base to the root tip.

#### 4.4.7. Green Leaf Area Measurement

The green leaf area is a core indicator of photosynthetic capacity, growth vigor, and anti-senescence potential. Measurement method: fixed-plant approach. In each plot, 10 representative plants were randomly selected (avoiding diseased or weak plants and edge plants). For each plant, 15 flag leaves were randomly selected (removing senescent or yellowed parts). The leaves were laid flat. Each leaf was scanned using a leaf area meter, and the green leaf area was recorded.

#### 4.4.8. Stay-Green Degree Measurement

Stay-green quantifies the capacity of leaves to retain green coloration and reflects plant senescence progression. This is a core morphological indicator. Measurement method: Stay green was calculated as the ratio of green leaf area at a given time to the maximum green leaf area.

Stay-green degree = (Green leaf area at a given time)/(Maximum green leaf area)

#### 4.4.9. Root Vitality Measurement

Root vitality is a core physiological indicator of the capacity of roots to absorb nutrients, tolerate stress, and delay senescence. Measurement method: *E. sibiricus* fibrous roots were selected (lignified primary roots were avoided). The samples were then rinsed with distilled water and blotted dry with filter paper. The samples were weighed (0.5 g) and cut into 1 cm segments. The segments were placed in stoppered glass test tubes. Then, 10 mL of 0.4% TTC solution and 10 mL of pH 7.0 phosphate buffer were added. The mixture was mixed thoroughly and incubated at 37 °C in the dark for 2 h (gently shaking once every 30 min). After incubation, 2 mL of 1 mol/L sulfuric acid was added. After the reaction was terminated, the supernatant was decanted, and the roots were rinsed several times with absolute ethanol (95%). Then, 20 mL of absolute ethanol (95%) was added to the tube. The formazan was extracted in an 80 °C water bath for 10 min. After cooling, the supernatant was collected and the absorbance (OD value) was measured at 485 nm. Calculation formula [40]:

Root vitality (mg/g·h) = (TTC reduction amount(mg))/(Root fresh mass(g) × incubation time(h))

#### 4.4.10. Chlorophyll Content Measurement

Chlorophyll content is a core physiological indicator of photosynthetic capacity, leaf senescence and stress tolerance. It is also a key indicator of the accumulation of photosynthates and the formation of forage yield. Measurement method: After heading in *E. sibiricus*, 10 single plants with similar vigor were randomly selected in each plot. Healthy flag leaves were selected. The veins were removed, cut into small pieces, and mixed thoroughly, to a weight of 50 mg. The samples were wrapped in aluminum foil and placed in a liquid nitrogen tank for transport to the laboratory to determine chlorophyll a, chlorophyll b, and chlorophyll a + b. The photosynthetic pigment content was determined using 95% ethanol extraction. The solution was extracted in the dark until the leaves became pale. The absorbance was measured using a UV spectrophotometer (SHIMADZU UV-2700, produced by Shimadzu Corporation, purchased via Shimadzu (China) Co., Ltd., Beijing, China) at 663 and 645 nm. The calculation formulas are as follows [41]:

Ca = 12.72 × D663 − 2.59 × D645

Cb = 22.88 × D645 − 4.76 × D663

CT = Ca + Cb = 20.29 × D645 + 8.05 × D663

Ca, chlorophyll a; Cb, chlorophyll b; CT, total chlorophyll.

#### 4.4.11. Soluble Sugar Content Measurement

Soluble sugars are the core products of plant carbon metabolism. These are key indicators of photosynthate accumulation, transport efficiency, stress tolerance, and anti-senescence capacity. Measurement method: A total of 0.2 g of fresh flag leaf sample (with the midrib removed and cut into small pieces) was weighed and placed in a mortar. Then, 5 mL distilled water was added, and the mixture was ground. The solution was transferred to a 100 mL volumetric flask. The samples were extracted in an 80 °C water bath for 30 min (shaken twice during extraction). After cooling, the solution was made up to 100 mL with distilled water, mixed thoroughly, and filtered. The supernatant was collected as the test solution (if turbid, the solution was centrifuged at 5000 rpm for 10 min). A total of 2 mL of test solution was pipetted into a 25 mL stoppered glass test tube. Then, 8 mL anthrone reagent was added. The mixture was mixed thoroughly and incubated in a boiling water bath for 10 min. After cooling, the absorbance (OD value) was measured at 620 nm. Finally, the soluble sugar content was calculated based on a standard curve [42].

#### 4.4.12. Soluble Protein Content Measurement

Soluble proteins are the primary forms of metabolic enzymes and stress-response proteins. It is a core indicator that directly reflects nitrogen accumulation, physiological activity, anti-senescence, and stress tolerance.

Measurement method: A total of 0.2 g of fresh flag leaf sample (with the midrib removed and cut into small pieces) was weighed and placed in a mortar. A total of 5 mL of phosphate buffer (pH 7.0) was added. The samples were then ground under liquid nitrogen (to prevent protein denaturation). The samples were transferred to a centrifuge tube. The samples were centrifuged at 12,000 rpm for 10 min. The supernatant was collected as the test solution. A total of 1 mL of test solution was pipetted into a 10 mL stoppered glass test tube and 5 mL of Coomassie Brilliant Blue reagent was added. The mixture was mixed thoroughly and allowed to stand for 5 min. The absorbance (OD value) was measured at 595 nm. Finally, the soluble protein content was calculated according to the standard curve [43].

#### 4.4.13. Photosynthetic Parameters Measurement

Photosynthetic parameters are core physiological indicators of photosynthetic efficiency, biomass production capacity and stress tolerance. Measurement method: selected clear-sky conditions. Between 9:00 and 11:00 a.m., the ultra-portable photosynthesis system LI-6800 was used at 0, 10, 20, 30, and 40 d after heading. For each experimental material, photosynthetic parameters were measured on the flag leaves of 10 pre-marked plants (continuously observed until the end of the experiment). The parameters included the net photosynthetic rate (Pn), transpiration rate (E), stomatal conductance (Gs), and inter-cellular CO_2_ concentration (Ci).

#### 4.4.14. Photosynthetic Decline Index

The photosynthetic decline index (PDI) is a core physiological indicator that quantifies the decrease in photosynthetic function. It also reflects the magnitude of the decline in photosynthetic efficiency during plant senescence. Calculation formula:

PDI = (Pn_0_ − Pnₜ)/(Pn_0_ × Δt) × 100%

where Pn_0_ is the net photosynthetic rate at the peak photosynthetic stage and Pn_t_ is the net photosynthetic rate at a given time, representing the number of days between the two time points. Interpretation: A positive PDI indicates a photosynthetic function decline, smaller values indicate a better stay-green capacity, and a negative value indicates an enhanced photosynthetic function.

### 4.5. Data Analysis

We performed preliminary data organization using Excel 2024. We then assessed normality (Kolmogorov–Smirnov) and homogeneity of variance (homogeneity) for plant height, stem diameter, water content, tiller number, root shoot ratio, primary root length, green leaf area, stay-green degree, root activity, chlorophyll content (chlorophyll a, chlorophyll b, chlorophyll a + b), soluble sugar content, soluble protein content, photosynthetic parameters (net photosynthetic rate, transpiration rate, stomatal conductance, inter-cellular CO_2_ concentration), and the photosynthetic decline index for normality (Kolmogorov–Smirnov) and homogeneity of variances (homogeneity). We then conducted multiple comparisons (LSD) within one-way ANOVA to evaluate significance at the 0.05 level using SPSS 19.0 (SPSS 19.0, Chicago, IL, USA).

We used the Mantel test to analyze correlations between phenotypic, photosynthetic, and physiological traits of *E. sibiricus* and leaf stay-green degree and the photosynthetic decline index. We applied mixed-effects models using the lme4 and glmm.hp packages to quantify the independent explanatory power of each factor for the leaf stay-green degree and photosynthetic decline index. We constructed a multi-criteria decision model, TOPSIS (Technique for Order Preference by Similarity to an Ideal Solution) with the plyr package, to comprehensively evaluate phenotypic traits and photosynthetic and physiological characteristics and to identify the most desirable stay-green *E. sibiricus* germplasm. We used Origin 2024 to compute the coefficients of variation and generate figures.

TOPSIS model: The TOPSIS model is a multi-criteria decision-making tool. It determines the best option by comparing the distance of each alternative to the ideal and anti-ideal solutions. It is widely used for evaluation and selection tasks.

The weighting procedure of the TOPSIS model with the entropy–weight method was executed in four steps (n = number of accessions, m = number of indicators).

Normalization–construct matrix P

p_ij_ = x_ij_/S_i_ x_ij_ (i = 1…n, j = 1…m)

Entropy value e_j_

q_ij_ = p_ij_/S_i_ p_ij_

e_j_ = −(ln n) − 1 S_i_(q_ij_ ln q_ij_)

Dispersion coefficient

d_j_ =1 − e_j_

Weight determination

w_j_ = d_j_/S_j_ d_j_ (S_j_ w_j_ = 1)

Weights were then applied to generate the weighted normalized matrix V = p_ij_ w_j_ for the subsequent TOPSIS ranking. The TOPSIS model was applied to evaluate *E. sibiricus* accessions. This includes data standardization of plant height, stem diameter, water content, tiller number, root shoot ratio, primary root length, green leaf area, stay-green degree, root activity, chlorophyll a, chlorophyll b, chlorophyll a + b, soluble sugar content, soluble protein content, net photosynthetic rate, transpiration rate, stomatal conductance, inter-cellular CO_2_ concentration, and the photosynthetic decline index. Subsequently, we constructed a weighted standardized matrix. The ideal and anti-ideal solutions were determined. Distances of each accession to both solutions were calculated. Finally, fitting degree coefficients (0–1, closer to 1 is better) were computed to identify the optimal germplasm resources.

## 5. Conclusions

We conducted a systematic three-year field study in Xihai Town on the phenotypic, photosynthetic, and physiological traits of stay-green *E. sibiricus*. We performed variance analysis, built mixed-effects models for correlation analysis, and applied the TOPSIS model to construct a comprehensive evaluation framework. We systematically assessed stay-green materials and screened the best germplasm. The main conclusions were as follows:

(1) Stay-green *E. sibiricus* germplasm was significantly superior to non-stay-green materials at phenotypic, photosynthetic, and physiological levels. After heading, as senescence progressed, stay-green materials exhibited slower rates of decline in phenotype, photosynthesis, and physiology than non-stay-green materials, and they retained larger green leaf area, higher chlorophyll content, photosynthetic capacity, osmotic regulators, and root activity in late senescence.
(2) Correlation analyses using the Mantel test and mixed-effects model indicated that the aboveground–underground (chlorophyll, root activity, soluble sugars, and photosynthesis) coordination mechanism (leaf–root coordination) ensures that root system activity guarantees underground nutrient supply, while chlorophyll stability maintains aboveground carbon fixation; together, these mechanisms govern the stay-green of *E. sibiricus.*
(3) Comprehensive evaluation using the TOPSIS model showed that the stay-green germplasm HB-15 had the best overall advantage. It was an ideal parent for breeding new stay-green varieties of *E. sibiricus* and an excellent material for ecological restoration of degraded grasslands.

## Figures and Tables

**Table 1:** Variation and genetic diversity of phenotypic and physiological traits associated with stay-green senescence in *E. sibiricus*.

Traits	Average	Standard Deviation	Coefficient Variation
Plant height (cm)	152.82	17.74	0.12
Stem diameter (mm)	2.42	0.74	0.31
Tiller number per plant	155.42	83.33	0.54
Moisture content	0.51	0.059	0.12
Root shoot ratio	0.36	0.076	0.21
Primary root length (cm)	32.16	5.99	0.19
Leaf green area (cm^2^)	13.39	6.96	0.52
Stay-green degree	0.86	0.028	0.032
Root vitality (mgTTC/g·h)	0.60	0.16	0.27
Soluble sugar content (mg/gFW)	14.75	2.18	0.15
Soluble protein content (mg/gFW)	15.07	2.46	0.16
Chlorophyll a (mg/gFW)	0.82	0.094	0.11
Chlorophyll b (mg/gFW)	0.56	0.071	0.13
Chlorophyll a + b (mg/gFW)	1.35	0.22	0.16
Photosynthetic rate (μmol CO_2_/m^2^·s)	13.46	2.55	0.19
Transpiration rate (mmol H_2_O/m^2^·s)	2.80	0.34	0.12
Stomatal conductance (mol H_2_O/m^2^·s)	0.25	0.065	0.26
Inter-cellular CO_2_ concentration (μmol/mol)	253.05	20.11	0.079
Photosynthetic decline index	0.012	0.0056	0.49

**Table 2:** Weight coefficients of indicators across years.

Traits	Weighting Coefficient			
	Second Year	Third Year	Fourth Year	Comprehensive Assessment
Plant height (cm)	0.055	0.056	0.059	0.056
Stem diameter (mm)	0.074	0.074	0.076	0.107
Tiller number per plant	0.052	0.145	0.051	0.090
Moisture content	0.097	0.097	0.088	0.097
Root shoot ratio	0.070	0.074	0.081	0.077
Primary root length (cm)	0.087	0.086	0.099	0.090
Leaf area (cm^2^)	0.121	0.050	0.098	0.061
Stay-green degree	0.047	0.050	0.049	0.051
Root vitality (mgTTC/g·h)	0.056	0.060	0.059	0.061
Soluble sugar content (mg/gFW)	0.052	0.057	0.053	0.056
Soluble protein content (mg/gFW)	0.063	0.069	0.065	0.069
Chlorophyll a (mg/gFW)	0.056	0.062	0.057	0.060
Chlorophyll b (mg/gFW)	0.046	0.049	0.046	0.049
Chlorophyll a + b (mg/gFW)	0.052	0.058	0.053	0.057
Photosynthetic rate (μmol CO_2_/m^2^·s)	0.059	0.077	0.059	0.066
Transpiration rate (mmol H_2_O/m^2^·s)	0.047	0.055	0.047	0.051
Stomatal conductance (mol H_2_O/m^2^·s)	0.047	0.049	0.047	0.049
Inter-cellular CO_2_ concentration (μmol/mol)	0.053	0.056	0.053	0.056
Photosynthetic decline index	0.047	0.051	0.046	0.050

**Table 3:** Basic information of the tested materials.

Germplasm	Latin Name	Phenotypic Attributes
HB-2	*E. sibiricus*	Characterized by robust culm, reduced leaf number, large panicle size, and extended green duration
HB-4	*E. sibiricus*	Characterized by profuse leafiness, compact panicle architecture, resistance to seed shattering, and extended green duration
HB-8	*E. sibiricus*	Characterized by profuse leafiness, large panicle size, susceptibility to seed shattering, and extended green duration
HB-10	*E. sibiricus*	Characterized by reduced leafiness, susceptibility to seed shattering, large panicle size, and extended green duration
HB-11	*E. sibiricus*	Characterized by susceptibility to seed shattering, reduced leafiness, small panicle size, and relatively extended green duration
HB-15	*E. sibiricus*	Characterized by profuse leafiness, resistance to seed shattering, increased plant height, and extended green duration
CK	*E. sibiricus* cv. Qingmu No. 2	Characterized by profuse leafiness, large panicle size, and rapid canopy senescence

## Data Availability

The data presented in this study are available on request from the corresponding author due to experimental project is still under confidentiality.

## Supplementary Materials

The following supporting information can be downloaded at: https://www.mdpi.com/article/10.3390/plants15132047/s1, Table S1: Basic information of the tested materials Appendix.
