# Supplementary material for: Phenotypic and Physiological Changes Associated with Senescence in Stay-Green Elymus sibiricus and Germplasm Screening
Source: Plants (Basel). 2026 Jul 1;15(13):2047. doi: 10.3390/plants15132047 (PMC13363980; doi:10.3390/plants15132047)
Supplement: Supplementary file 1 [file plants-15-02047-s001.zip › plants-4305702-supplementary.pdf]

Table S1. Basic information of the tested materials Appendix

**Gemplasm**	**Plant height (cm)**	**Stem diameter (mm)**	**Tillering number of per plant**	**Leaf area (cm^2)**
HB-2	156.29	2.11	127.77	12.46
HB-4	133.68	2.69	138.72	11.18
HB-8	164.27	2.72	217.35	16.71
HB-10	158.89	2.13	131.52	13.85
HB-11	163.00	2.27	150.06	15.28
HB-15	167.54	3.03	212.94	18.07
CK	126.55	2.03	109.31	6.27
